# The association between academic engagement and achievement in health sciences students

**DOI:** 10.1186/1472-6920-13-33

**Published:** 2013-02-27

**Authors:** Maria J Casuso-Holgado, Antonio I Cuesta-Vargas, Noelia Moreno-Morales, Maria T Labajos-Manzanares, Francisco J Barón-López, Manuel Vega-Cuesta

**Affiliations:** 1 Faculty of Health Sciences at University of Malaga, Malaga, Spain; 2 School of Clinical Sciences of the Faculty of Health at the Queensland University Technology, Brisbane, Australia

**Keywords:** Academic achievement, Academic engagement, Health sciences students, University education, Cross sectional study

## Abstract

**Background:**

Educational institutions play an important role in encouraging student engagement, being necessary to know how engaged are students at university and if this factor is involved in student success point and followed.

To explore the association between academic engagement and achievement.

**Methods:**

Cross-sectional study. The sample consisted of 304 students of Health Sciences. They were asked to fill out an on-line questionnaire. Academic achievements were calculated using three types of measurement.

**Results:**

Positive correlations were found in all cases. Grade point average was the academic rate most strongly associated with engagement dimensions and this association is different for male and female students. The independent variables could explain between 18.9 and 23.9% of the variance (p < 0.05) in the population of university students being analyzed.

**Conclusions:**

Engagement has been shown to be one of the many factors, which are positively involved, in the academic achievements of college students.

## Background

Student engagement in post-school education has been researched since the 1990s, have been considered as an important factor in determining student learning and personal development during college [1,2].

The concept of engagement in relation to the student university experience is generally acknowledged as a multidimensional phenomenon that may result from a variety of factors relating to the individual and the context in which they are learning [3]. Researchers have proposed a variety of different ways to describe student engagement, indicating the complexity of this concept. It has at least two general meanings [4]. One emphasizes the degree of willing student compliance with organisational and subject rules, values and processes. The other focuses on students’ active participation and emotional commitment to their learning. It is the second meaning that underpins this paper.

There is growing recognition of the importance of understanding student engagement and the problem of disengagement in tertiary institutions. Investigating factors affecting engagement and disengagement can provide insights into student performance, progression and retention. Assessment of engagement is potentially useful when evaluating the quality of student learning experiences and making decisions about resource provision, course content and delivery [5].

In this way, it can be highlighted *The National Survey of Student Engagement* (*NSSE*), which has been used in USA and Canada since 2000 [6], and *The Australian Survey of Student Engagement* (AUSSE), which has been used in Australian and New Zealand institutions since 2007 [7]. They are the largest educationally focused cross-institutional surveys in those countries.

Regarding student engagement, educational institutions have an important role [8]. XXI century students are considerably diverse in backgrounds, personalities, and learning styles [9], but teachers should consider what it is that motivates students to become engaged and should use these findings to improve student engagement with University work [10].

Engaged students are more able to cope with academic stress [11], and are more satisfied [12,13], which perhaps will lead in the future to more professionals having a sense of well-being and less feeling burned out, a syndrome which traditionally has been dealt with by health care professionals [14].

As regards students in Health Sciences, most of the research refers to nursing students [11,15,16] and generally focuses on analyzing different ways of teaching and the use of new educational tools. Key findings indicate that engagement is positively associated with more active learning and student participation [17-24] and with the use of the new technologies in teaching such as audience response systems (e.g. clickers) [25-28] YouTube videos [29,30] or special digital games [31] .

Internationally, universities are interested in measuring the learning outcomes of their students. The association between engagement and academic achievement in college students has not been researched in enough detail. Although some researchers have observed a positive relationship [32-35] and others have not [36-39], we believe that it is necessary to examine the issue of engagement in post-compulsory education more in depth.

This paper focuses on the association between engagement and academic achievement in Health Sciences students. We hypothesize that the more engaged students will be more likely to have the best academic achievement.

## Methods

### Design and participants

We conducted a cross-sectional research at the University of Malaga (Spain). Health Sciences students were eligible for participation if they were enrolled in Nursing, Physiotherapy, Podiatry or Occupational Therapy studies during the academic year 2010/11.

### Recruitment

We used a convenience sample and tried to recruit all participants (n = 911) by approaching them in class and asking them to complete the online survey. The response rate was 35.6% (N = 324). 6.2% of the surveys were removed from the sample due to missing data. 37.5% of the total sample studied Nursing, 26% Physiotherapy, 13.5% Podiatry and 23% Occupational Therapy. Written informed consent was obtained from the students in accordance with the Helsinki Declaration (2000 modification). This study had ethics approval from the Research Ethics Committee of the Faculty of Nursing, Physiotherapy, Podiatry and Occupational Therapy of the University of Malaga (Spain).

### Measurement instruments

The variables obtained by mail were: age, gender, degree, academic year, means and priority of admission. We also obtained engagement scores.

#### Utretch work engagement scale for students (UWES-S)

The measurement of academic engagement was determined by using the Utrecht Work Engagement Scale for Students (UWES-S). The questionnaire was originally created for Dutch students and was adapted to a Spanish setting after cross-cultural research with Dutch, Portuguese and Spanish university students [40].

The Spanish version has 14 items, featuring scores ranging from 0 “Never” to 6 “Always”. The Spanish version has 14 items, featuring scores ranging from 0 “Never” to 6 “Always”. An exploratory factorial analysis of the scale was conducted. Kaiser-Meyer-Olkin measure was 0.846 and Bartlett’s test 0.000. The method of varimax rotation showed a three-dimensional structure (vigour, dedication and absorption) explaining 65.26% of the total variance. Correlations between items were also tested. Similar data has been reported by various authors (Table 1) [41]. The reliability of each dimension demonstrated a good internal consistency, with Cronbach´s coefficients of 0.74, 0.87 y 0.84 respectively.

**Table 1:** Statements in single item measure of engagement (UWES-S)

**Engagement dimensions**	
**Response number**	**Statement**
**Vigor**	
1	When I’m studying, I feel mentally strong
2	I can continue for a very long time when I am studying
3	When I study, I feel like I am bursting with energy
4	When studying, I feel strong and vigorous
5	When I get up in the morning, I feel like going to class
**Dediction**	
1	I find my studies to be full of meaning and purpose
2	My studies inspire me
3	I am enthusiastic about my studies
4	I am proud of my studies
5	I find my studies challenging
**Absorption**	
1	Time flies when I’m studying
2	When I am studying, I forget everything else around me
3	I feel happy when I am studying intensively
4	I can get carried away by my studies

Information about students’ admission scores, the subjects they were enrolled in, exams, qualifications, etc. were obtained from students’ transcripts. Using this data, researchers manually calculated three rates for each student, as explained below.

#### Academic achievement

Academic achievement has been mainly measured with reference to the grade point average –GPA- [33,35,42-45]. In this study, this variable was more widely performed by the calculation of three individual rates: success rate (SR), performance rate (PR) and grade point average (GPA). In order to have a testable data, the GPA was only calculated if student had passed at least 70% of the initial credits after enrolment. PR and SR can have values from 0 to 1. GPA can range from 0 to 4.

The way each rate was calculated is shown below:

▪ SR = number of passed credits/ total number of credits taken in exam × 100.

▪ PR = number of passed credits/ total number of enrolled credits × 100.

▪ GPA = Σnumber of credits of a subject × grade / total number of credits taken in exam.

It is necessary to highlight the differences between SR and PR because they seem to be very similar but they have different interpretations. When we speak about SR, we are assessing how successful are students in their exams, without taking into account the total success in one course. On the other hand, when we speak about PR we are assessing a broader success that refers to the total enrolled credits and not only to credits taken in exam.

### Setting and procedure

In May 2011 a web application containing sociodemografic variables and UWES-S scale was sent to registered students’ email addresses by researchers, being possible to complete this information during the whole month of May. Data on academic achievement (students admission scores, the subjects they were enrolled in, exams, qualifications, etc.) were collected from the student administrative database. Using these data, researchers manually calculated three rates for each student, as explained above.

Two datasets were then anonymised further by the encryption of students’ civil registration numbers, using new unique identification numbers. The researchers complied fully with established ethical rules.

### Data analysis

Descriptive statistics were obtained by measuring the central tendencies and rate of dispersion of the variables studied. The main analysis was guided towards a search for the correlations between engagement dimensions (vigour, dedication and absorption) and academic achievement (SR, PR and GPA). We used Pearson’s linear correlation with CI 95% and a multivariate correlation. We looked for simple and multiple regression models. We used SPSS for Windows V 15.0.

## Results

In total, 304 students were analyzed. The distribution of the sample and descriptive statistics for each engagement dimension and academic results for the groups are shown in Table 2. Mean differences were tested taking into account gender (T-test) and degree (ANOVA). Regarding engagement dimensions, there were no significant differences between genders (*Vigor*: F_2,257_ = 0.019;p = 0.214. *Dedication*: F_2,257_ = 0.786;p = 0.090. *Absorption*: F_2,257_ = 1.579;p = 0.075) or academic degrees (*Vigor*: F_3,255_ = 0,153;p = 0,928. *Dedication*: F_3,255_ = 2,061;p = 0,106. *Absorption*: F_3,255_ = 0,285;p = 0,836). However, different degrees differed significantly in terms of their success rate (F_3,285_ = 0,66; p = 0,027), performance rate (F_3,285_ = 0,36; p = 0,000) and grade point average (F_3,223_ = 0,672; p = 0,03). Nursing students seems to have the best academic achievement.

**Table 2:** Descriptive characteristics of the sample (n = 304)

**Variable (Unit)**	**Average ± SD**^**a**^					
	**Women**	**Men**	**Nursing**	**Physiotherapy**	**Podiatry**	**Occupational Therapy**
n	236 (77.6%)	68 (22.4%)	114 (37.5%)	79 (26%)	41 (13.5%)	70 (23%)
Age (years)	21.94 ± 5.27	22.56 ± 6.07	21.97 ± 6.18	21.40 ± 4.65	25.32 ± 6.47	21.16 ± 3.44
**Engagement dimensions (scale 0 to 6)**						
Vigor	3.9 ± 1.01	3.28 ± 1.08	3.13 ± 1.06	3.19 ± 1.03	3.12 ± 1.06	3.06 ± 0.91
Dedication	4.95 ± 1.01	4.67 ± 1.23	4.87 ± 1.18	4.83 ± 1.06	4.61 ± 0.97	5.17 ± 0.86
Absorption	3.07 ± 1.24	3.42 ± 1.40	3.07 ± 1.36	3.26 ± 1.87	3.13 ± 1.31	3.16 ± 1.27
**Academic achievements**						
Success rate (scale 0 to 1)	0.95 ± 0.55	0.94 ± 0.11	0.94 ± 0.12	0.89 ± 0.17	0.91 ± 0.16	0.87 ± 0.14
Performance rate (scale 0 to 1)	0.81 ± 0.21	0.86 ± 0.17	0.88 ± 0.18	0.80 ± 0.21	0.77 ± 0.19	0.76 ± 0.17
Grade Point Average (scale 1 to 4)	1.97 ± 0.37	2.04 ± 0.40	2.05 ± 0.41	1.99 ± 0.30	1.75 ± 0.31	2.00 ± 0.39

a: Standard deviation.

A Pearson’s linear correlation analysis of the variables is presented in Table 3. Positive correlations are found in all cases. Although vigor appeared to be the most significant dimension, the size of the effect in all cases can be described like small [46].

**Table 3:** Pearson correlation coefficient between engagement dimensions and academic results

**Related variables**	**Correlation index (CI)**		
***Engagement dimensions***	***Academic achievement rates***		
	**SR**^**a**^	**PR**^**b**^	**GPA**^**c**^
Vigor	0.150*	0.160*	0.221**
Dedication	0.110	0.096	0.137
Absorption	0.093	0.033	0.160*

a: Succes rate.

b: Performance rate.

c: Grade point average.

* = p < 0.05.

** = p < 0.01.

Table 4 indicates that GPA was the academic rate most strongly associated with engagement dimensions and that this association was different for male and female students. The highest effect in women appeared in the dedication dimension; whereas with male students the vigor dimension showed the highest effect with relation to a medium- sized case like this [46].

**Table 4:** Pearson correlation coefficient between engagement dimensions and academic results by gender

**Related variables**	**Female CI**			**Male CI**		
***Engagement dimensions***	***Female academic achievement rates***			***Male academic achievement rates***		
	**SR**^**a**^	**PR**^**b**^	**GPA**^**c**^	**SR**^**a**^	**PR**^**b**^	**GPA**^**c**^
Vigor	0.151*	0.132	0.119	0.266	0.238	0.503**
Dedication	0.124	0.116	0.211**	0.119	0.097	−0.053
Absorption	0.086	−0.006	0.157	0.248	0.119	0.140

a: Succes rate.

b: Performance rate.

c: Grade point average.

* = p < 0.05.

** = p < 0.01.

The results of multiple regression analysis suggest that the independent variables (vigor, dedication and absorption) could explain between 18.9 and 23.9% of the variance (p < 0.05) in the population of university students analyzed. None of the social variables specified retained statistical significance as independent predictors of academic achievements (Table 5).

**Table 5:** Multiple regression analysis with the same predictor over 3 dependent variables about academic achievement

**Depedent Variable**	**Predictor Variables**	**Untandardized Beta ± Std error (*p*)**	**R**
SR^a^	Vigor	.046 ± .027(p = 0,083)	0.166 (p = 0.081)
	Dedication	.025 ± 0.23(p = 0,270)	
	Absorption	−003 ± 0.22(p = 905)	
PR^b^	Vigor	.038 ± .015 (.011)*	0.189 (p = 0.033)*
	Dedication	015 ± 013(.239)	
	Absorption	−016 ± 012(.182)	
GPA^c^	Vigor	070 ± 031(.027)*	0.239 (p = 0.010)**
	Dedication	032 ± 0.27(.239)	
	Absorption	003 ± 026(.912)	

a: Succes rate.

b: Performance rate.

c: Grade point average.

* = p < 0.05.

** = p < 0.01.

## Discussion

This study has analyzed the association between engagement and academic achievement in Health Sciences students. After multiple analyses, significant associations were found, although the relationships were not sufficiently strong to have a high predictive value (r < 0.3).

The main finding of this research is that in virtually all cases a positive correlation between the three dimensions of academic engagement and the three achievement rates analyzed has been observed. The GPA seems to be the variable most associated with engagement dimensions (r = 0.221; p < 0.01). It has also been observed as a factor in differences between genders. Female GPA is mainly associated with dedication (r = 0.211; p < 0.01), while male GPA does with vigor (r = 0.503; p < 0.01) .Into the engagement dimensions, the vigor showed the best level of contribution into the different regression model in each academic achievement.

The present data are consistent with previous findings. Kuh, Cruce, Dhoup y Kinzie [33] also observed a positive correlation between engagement, GPA and the chances of returning for a second year at college. Svanum & Bigatti [34] demonstrated that highly academically engaged students were 1.5 times more likely to graduate and required approximately 1 semester less to do so, also obtaining a higher GPA. It has also been observed that students who leave college prematurely are less engaged than their counterparts who persist [47].

In a similar way, SR was slightly associated with three engagement dimensions [39] and with vigor and absorption by Manzano [37,38]. On the other hand, Bresó and Gracia [32] also observed that engagement dimensions related to academic effectiveness.

A second finding of this study is the fact that engagement could explain between 19.5-23.9% of the variance in academic achievement. However, there are important differences for each rate. In the case of SR, any engagement dimensions have a predictive value. These results support the findings of Martínez and Salanova [39], who observed that engagement had a predictive value only for student drop out intentions, but not for success with studies.

On the other hand, vigor could explain 19.5% of the total variance of PR and 23.9% of the total of GPA. The dedication and absorption dimensions were not statistically significant to have a predictive value for these rates.

The present results suggest that it is necessary to think about the fact that academic engagement may perhaps relate in a higher way to other qualitative constructs like satisfaction with studies [12], life satisfaction [13], self-efficacy beliefs [35], happiness [35] or dropping out of studying. It could be possible that associations with quantitative variables, as measured in this research, are not so strong.

However, we cannot forget that when we analyse academic achievement there are many personal, educational and contextual variables that have also an important role. That is to say, student success can be considered to be a multivariate factor. For this reason, we think that it is really difficult to isolate variables that explain big variances. In this way, we think the major implication of this study is the fact that our results have proved that academic engagement is one of those many factors that can have a positive influence on the academic achievement of students.

This could be a useful way for teachers in post-compulsory education to consider what more they can do to engage students in their learning Teachers who are conscious of the context within which they work and the backgrounds of their students, and who enquire about the motivational needs of their students, have a better chance of engaging them than those who do not. Interacting with staff is one of the most powerful learning activities in which students engage [1,2,48].

### Study limitations

This study has some limitations that must be taken into account when interpreting the findings. Firstly, a cross-sectional study is not the best way to establish causality between factors, but can set up associations that help guide us towards further study. In order to solve this question, we are currently working on a four year longitudinal study with a cohort of the same students already tested. Secondly, the use of a web application may have influenced the response rate.

On the other hand, it is true that no previous researchers have analysed the relationship between academic engagement and student achievement in the way we have done, by using three individual rates that can be generalized to any case to give us more information.

## Conclusions

We hypothesized that the more engaged students would be more likely to have the best academic achievement. Low correlation scores do not allow us to asseverate this fact, but our results have proved that academic engagement is one of many factors that have positively influenced the academic achievement of the students tested.

Although the relationships found were not sufficiently strong to have a high predictive value, they suggest that the more engaged students are more likely to have the best academic achievements.

However, it is necessary to explore this relationship more extensively, by including other academic variables like satisfaction, self-efficacy, ways of learning, etc. that can help us to understand the ways in which college students achieve greater or lesser degrees of academic success.

Finally, these findings also suggest that student affairs practitioners should assess and emphasize academic engagement as one significant and important component in a successful college career. It is necessary to ask yourself: What is important in motivating students to engage and how frequently is that motivation used in practice?

### Ethical approval

This research was approved by The Research Ethics Committee of the Faculty of Nursing, Physiotherapy, Podiatry and Occupational Therapy at the University of Málaga.

## Abbreviations

AUSSE: Australian survey of student engagement; GPA: Grade point average; NSSE: National survey of student engagement; SR: Success rate; PR: Performance rate; UWES-S: Utretch work engagement scale for students.

## Competing interests

The authors declare that they have no competing interests. The authors alone are responsible for the content and writing of the article.

## Authors’ contributions

All the authors have made significant contributions to the article. AIC-V and MTL-M coordinated the project and contributed to the conception and design of this study. MJC-H was responsible of the acquisition of data, screened articles and drafted the manuscript. AIC-V reviewed and edited the manuscript and contributed to the analysis and interpretation of data. FJB-L coordinated the administration of web surveys, checked data extraction and the statistical analysis. NM-M was also responsible of the acquisition of data. MV-C collaborated in the acquisition of data and in the calculation of the academic rates. All authors read and approve the final manuscript.

## Author’s information

MJC-H is Post doc fellow of the Physiotherapy Department at the University of Malaga, Spain. AIC-V is a Professor of Physiotherapy at the University of Málaga, Spain and Adjunct Associate Professor of the School Clinical Science at Queensland University of Technology, Australia and FJB-L is Professor of Preventive Medicine at the University of Malaga, Spain. NM-M is Professor of Physiotherapy at the University of Málaga, Spain. MTL-M is the Dean of Faculty of Health Sciences at the University of Malaga, Spain. MVC is PhD Candidate at the University of Malaga, Spain.

## Pre-publication history

The pre-publication history for this paper can be accessed here:

http://www.biomedcentral.com/1472-6920/13/33/prepub
